# A rare left ventricular cardiac myxoma mimicking fibroma

**DOI:** 10.1186/s13019-022-01968-7

**Published:** 2022-08-26

**Authors:** Liang-Yan Xia, Hong-Ling Zhu, Rong-Hang Li, Xiao-Hua Pan, Bo Liu, Jing Xu

**Affiliations:** 1 Department of Stomatology, The First Hospital of Jilin University, Changchun, 130021 Jilin China; 2 Department of Orthopedic Surgery, The Second Hospital of Jilin University, Changchun, 130021 Jilin China; 3 Department of Ultrasound, The First Hospital of Jilin University, Changchun, 130021 Jilin China

**Keywords:** Myxoma, Fibroma, Echocardiography, Computed tomography

## Abstract

**Background:**

In most cases, it is not difficult to differentiate common left ventricular (LV) cardiac myxomas from fibromas because they are different disease entities and have different imaging findings. Herein, we present a case of a tumor with histological characteristics of a LV cardiac myxoma even though its imaging and macroscopical views were similar to that of fibroma.

**Case presentation:**

A 65-year-old woman was admitted to the hospital with chest tightness and palpitations which persisted for 2 years. Transthoracic echocardiogram and transesophageal echocardiography revealed a 23 mm × 8 mm, polyp-like-shaped, homogeneous, firm, solitary, mobile and solitary LV mass, which protruded into the left atrium during systole, resulting in mild mitral regurgitation. LV contrast-enhanced echocardiography revealed that there was little contrast agent filling in the LV mass. To further clarify the nature of the mass, non-enhanced and contrast-enhanced coronary computed tomography (CT) angiograms showed a 19 mm × 8 mm relatively homogeneous low density with punctate calcifications mass and no significant enhancement. Thus, we preoperatively diagnosed her condition as a LV fibroma and performed excision of the tumor under cardiopulmonary by-pass by using port-access approach through right mini-thoracotomy. The postoperative pathological diagnosis of the tumor was in fact a LV myxoma.

**Conclusions:**

LV cardiac myxomas mimicking fibroma makes diagnosis difficult, and sonographers should be aware of this imaging changes.

## Background

Myxomas are the most common benign tumor of the heart, about 72% to 92% of myxomas are located in the left atrium [1, 2]. Left ventricular (LV) myxomas are very rare, comprising only 0.7% to 3.6% of all cardiac myxomas [3]. Cardiac fibromas are primarily detected in infants and children but are occasionally reported in adults, and are usually located on one of the LV free walls [4].

Their early diagnosis is difficult since the symptoms and signs may be nonspecific, and symptomatic patients may present with a wide range of symptoms, including chest pains, palpitations, episodes of syncope, or nonspecific discomfort [5].

In general, there is little confusion in differentiation of LV cardiac myxomas and fibromas as they have different imaging manifestations [6, 7]. In the present report we describe a case of mitral regurgitation caused by LV cardiac myxoma that was misidentified as fibroma via imaging examination preoperatively, and excised with the right mini-thoracotomy approach.

## Case presentation

A 65-year-old woman was admitted to the hospital with chest tightness and palpitations which persisted for 2 years. The patient had a free past medical history, and denied any other relevant personal or family history. In physical examination, there were no remarkable signs, and her vital signs were normal. And, there was no hematological and biochemical disorders. The levels of tumor biomarkers were normal.

**Fig. 1:**
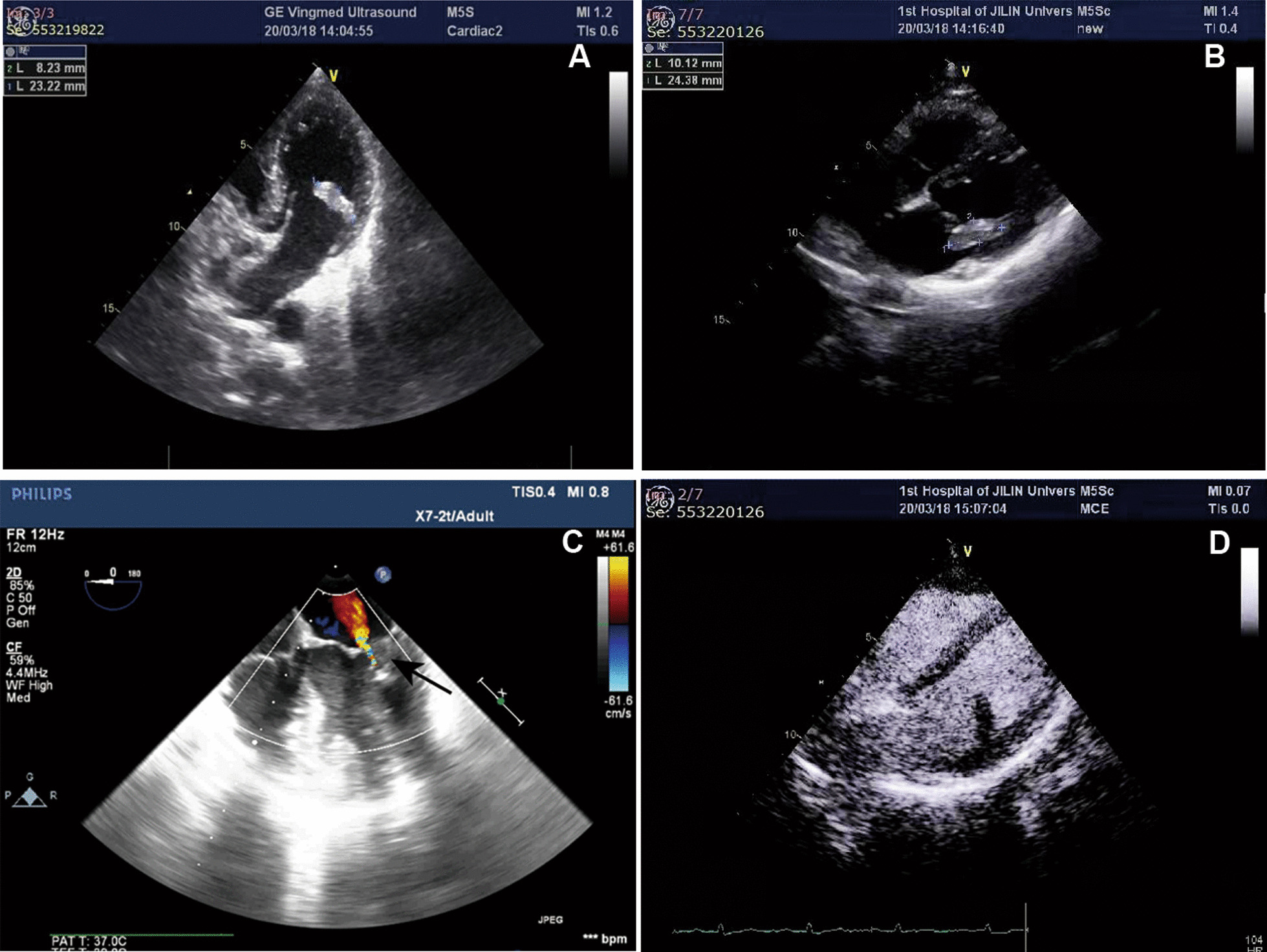
Ultrasound of the cardiac tumor. **A** Two-chamber view in transthoracic echocardiography shows a 23 mm × 8 mm mass which was polyp-like-shaped, homogeneous, firm, mobile and solitary centered on the lateral wall of the left ventricle. **B** Grey and **C** Color doppler flow imaging of four-chamber view in transesophageal echocardiography shows that the mass had regular margins and was pedunculated, with a stalk originating from the LV wall, and the mass protruded into the left atrium during systole, resulting in mild mitral regurgitation. **D** View of left ventricular contrast-enhanced echocardiogram shows little contrast agent filling

**Fig. 2:**
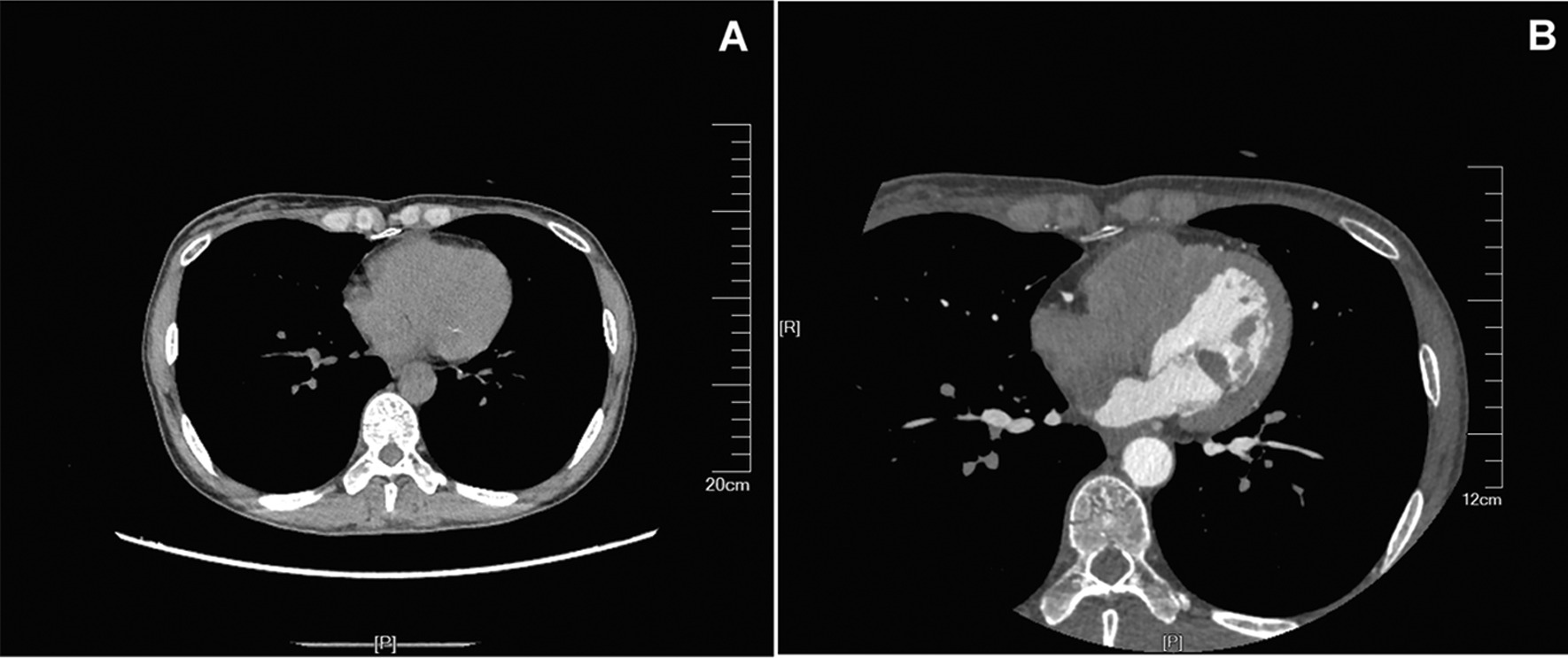
**A** Chest CT revealing a calcified mass lesion in the left ventricle. **B** Contrast-enhanced coronary CT angiograms revealing a homogeneous low density mass with punctate calcifications measured 19 mm × 8 mm and no significant enhancement

Transthoracic echocardiogram (TTE) revealed a 23 mm × 8 mm mass which was polyp-like-shaped, homogeneous, firm, mobile and solitary (Fig. 1A). Further transesophageal echocardiography (TEE) revealed that the mass had regular margins and was pedunculated, with a stalk originating from the LV wall, and the mass protruded into the left atrium during systole, resulting in mild mitral regurgitation (Fig. 1B and C). To further clarify the nature of the mass, LV contrast-enhanced echocardiography was performed. And there was very little contrast agent filling in the LV mass (Fig. 1D). Her chest non-contrast computed tomography (CT) showed a calcified mass lesion in the left ventricle (Fig. 2A). The contrast-enhanced coronary CT angiograms demonstrated a 19 mm × 8 mm mass centered on the lateral wall of the left ventricle. The mass was relatively homogeneous low density with punctate calcifications and no significant enhancement (Fig. 2B). The preoperative diagnosis was the cardiac fibroma.

**Fig. 3:**
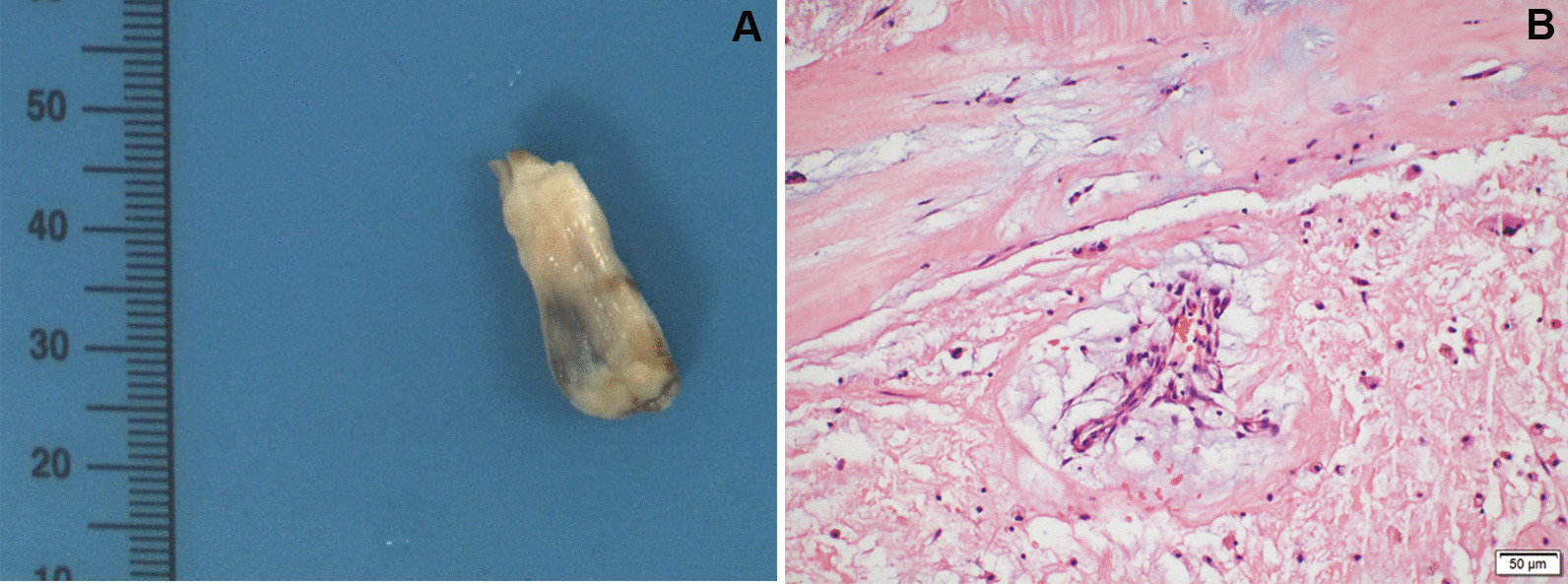
Images of cardiac mass specimen. **A** The outer surface was gray-white, firm, polyp-like. **B** showed irregular tumor cells surrounded with voids and scattered with interstitial sparseness (hematoxylin and eosin stain, × 400)

Excision of the tumor under cardiopulmonary bypass was performed adopting a port-access approach through the right mini-thoracotomy. After incision of the left atrium, no thrombosis was detected. Then the mitral valve was pulled open, and a mass of gray-white, firm, polyp-like, was seen. The tumor was connected to the posterior wall of the LV and did not invade the heart muscle, and was completely excised with an adequate margin of endocardium (Fig. 3A). After re-exploration for no other mass, the surgeon sutured layer by layer and ended the operation. Postoperative histopathological examination showed that the tumor cells were irregular, surrounded with voids, and scattered with interstitial sparseness, which confirmed the cardiac myxoma (Fig. 3B).

After tumor removal, the patient was free of chest tightness and palpitations and recovered well. At 9-day after the surgery, TEE showed that the cardiac mass and mitral regurgitation resolved completely. In the 3-year follow-up period, this patient experienced no tumor recurrence.

## Discussion and conclusions

Cardiac myxoma is the most common primary cardiac tumor. Most cardiac myxomas are located in the left atrium, and LV myxomas are quite rare [8]. Unlike myxomas, cardiac fibromas usually arise in the LV free wall [9].

Their early diagnosis is difficult since the symptoms and signs may be nonspecific, and symptomatic patients may present with a wide range of symptoms, which are determined by tumor location, size, and embolization tendency [10]. Pain, palpitations, episodes of syncope, systemic embolization and its complications are the main symptoms of LV myxomas [11]. In our case, the patient only presented with chest tightness and palpitations, and without other special symptoms.

Myxomas are not typically detected during physical examinations or in laboratory tests. Echocardiography, CT and magnetic resonance imaging (MRI) are the imaging modality enabling an accurate myxoma diagnosis [2]. The typical echocardiographic features of myxoma present as a solitary, heterogenous, slightly mobile, spherical mass attached to the surface of the endocardium, with a wide pedicle. What is more, internal hypoechoic areas, spot-like echogenic foci and lobular surface protrusions can be sometimes seen in myxomas [12, 13]. Cardiac fibroma always manifested as a homogeneous, solid, firm or rubbery, solitary mass, with a size range from 2 to 10 cm [4, 7]. They may display well circumscribed or infiltrating margins, and are usually located on one of the LV free walls [4]. Compared with myocardium in LV contrast-enhanced echocardiography, more contrast media was observed in the myxoma mass, only scattered and dotted contrast agent was present in the fibroma mass as it is mainly composed predominantly of collagen in adults [7, 14, 15].

In non-enhanced and contrast-enhanced CT, myxoma usually present as a heterogeneously low attenuated mass in the heart chamber with a smooth, irregular or villous surface, and heterogeneous enhancement [16, 17]. Cardiac CT often demonstrates a homogeneous low density mass with partial calcifications in fibromas, whereas there was no imaging of contrast agent within it [18, 19]. Echocardiography and CT findings of this case suggest a cardiac fibroma at the imaging level.

Pathology is the gold standard for diagnosing heart tumors. Macroscopically, cardiac myxoma typically present as a single, pedunculated, fragile, and irregular shaped lesion with an intact capsule, whereas cardiac fibroma is solitary, circumscribed, firm, gray-white, partially calcified neoplasms without a capsule[20]. Histologically, cardiac myxoma is characterized by irregular or star shaped cells loosely dispersed within a mucoid ground substance [21]. However, cardiac fibroma is composed predominantly of collagen in adults [7].In our case, the surgical specimen, which is gray-white, firm, polyp-like, and composed by irregular tumor cells surrounded with voids and scattered with interstitial sparseness, is similar to cardiac fibroma at macroscopical level, but confirmed the cardiac myxoma at histological level.

Surgery is one of the most effective treatments for cardiac myxoma. And a complete surgical resection is highly recommended as the recurrence of cardiac myxoma may be caused by incomplete tumor removal [22]. Furthermore, regular follow-up with TTE is necessary to detect possible recurrences of this kind of cardiac tumor. In the 3-year follow-up, our patient had no recurrence of the tumor.

In conclusion, LV cardiac myxomas mimicking fibroma makes diagnosis difficult, and sonographers should be aware of this imaging changes.

## Data Availability

All data generated or analysed during this study are included in this published article.

## Abbreviations

LV: Left ventricular
TTE: Transthoracic echocardiography
TEE: Transesophageal echocardiography
MRI: Magnetic resonance imaging
CT: Computed tomography
